# Supplementary material for: Identification of photoperiod-induced specific miRNAs in the adrenal glands of Sunite sheep (Ovis aries)
Source: Front Vet Sci. 2022 Jul 22;9:888207. doi: 10.3389/fvets.2022.888207 (PMC9354845; doi:10.3389/fvets.2022.888207)
Supplement: Supplementary file 1 [file Data_Sheet_1.ZIP › Supplementary file/miRDeep2 file.zip!/miRDeep2 file/Novel_Structure/SP42A2/Novel_416.pdf]

Provisional ID	: NC_019458.2_341
Score total	: 2.2
Score for star read(s)	: -1.3
Score for read counts	: 0
Score for mfe	: 1.9
Score for randfold	: 1.6
Score for cons. seed	:
Total read count	: 42
Mature read count	: 42
Loop read count	: 0
Star read count	: 0

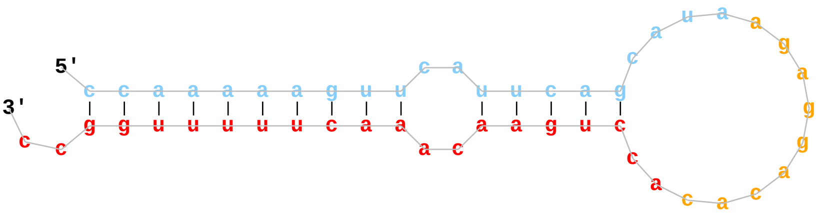

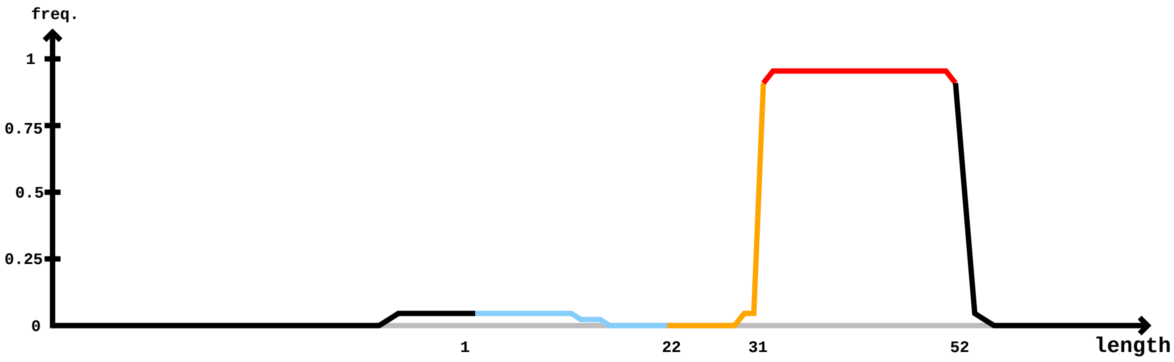

Star Mature

```
5'- aucacauuagugaagugcuaucccauagaaauauuggguuggccaaaaaguucauucagcauaagaggacacaccugaacaaacuuuuuggccaacccaguauaaagcaagc   -3'    exp
    .((((....))))..((((...........((((((((((((((((((((((..(((((...............)))))..))))))))))))))))))))))..))))...     reads      mm         sample
    .................................uuggAuuggccaaaaaguuc...........................................................     1          1          seq
    ..................................uggguuggccaaaaCguucauu........................................................     1          1          seq
    ......................................................................acaccugaacaaacuuuuuggc....................     2          0          seq
    ........................................................................aUcugaacaaacuuuuuggcc...................     25         1          seq
    ........................................................................accugaacaaacCuuuuggcc...................     1          1          seq
    ........................................................................aUcugaacaaacuuuuuggcca..................     11         1          seq
    ........................................................................accugaacaaacCuuuuggccaac................     1          1          seq
    .........................................................................ccugaacaaCcuuuuuggcc...................     1          1          seq
    .........................................................................Ucugaacaaacuuuuuggccaa.................     1          1          seq
```
